# Effect of cold plasma synergistic ultrasound-assisted enzyme treatment on the improvement of hygroscopicity and quality characteristics of jujube powder

**DOI:** 10.1016/j.ultsonch.2025.107568

**Published:** 2025-09-11

**Authors:** Chenlu Zeng, Yuxing Liu, Hao Dong, Yaxuan Liao, Ziyi Wang, Wenjing Yang, Jinling Lei, Guogang Chen, Wanting Yang, Shaobo Cheng

**Affiliations:** a College of Food Science and Technology, Shihezi University, Shihezi 832000, China; b Engineering Research Center of Storage and Processing of Xinjiang Characteristic Fruits and Vegetables, Ministry of Education, School of Food Science and Technology, Shihezi University, Shihezi, Xinjiang 832000, China; c Key Laboratory of Characteristics Agricultural Product Processing and Quality Control (Co-construction by Ministry and Province) Ministry of Agriculture and Rural Affairs, School of Food Science and Technology, Shihezi University, Shihezi, Xinjiang 832000, China; d Key Laboratory for Food Nutrition and Safety Control of Xinjiang Production and Construction Corps, School of Food Science and Technology, Shihezi University, Shihezi, Xinjiang 832000, China; e Shihezi Testing Institute of Quality and Metrology, Shihezi 832000, China

**Keywords:** Cold plasma, Ultrasound, Glucose oxidase, Hygroscopicity, Desugarized jujube powder

## Abstract

This study aimed to obtain high-quality jujube powder by reducing its hygroscopicity and tendency to agglomerate. Jujube slices were pretreated with enzyme treatment (EZ), ultrasound-assisted enzyme treatment (US-EZ), cold plasma-assisted enzyme treatment (CP-EZ), and a combined cold plasma synergistic ultrasound-assisted enzyme treatment (CP-US-EZ) prior to vacuum microwave drying (MVD). Given that glucose and fructose are key contributors to hygroscopicity and caking, the impact of each pretreatment on the desugarization effect, nutritional quality, and physical properties of the resulting powder was evaluated. All four pretreatments effectively reduced sugar levels and decreased the agglomeration phenomenon, with CP-US-EZ showing the most pronounced effect. Structural analysis via scanning electron microscopy (SEM) and X-ray diffraction revealed that CP and US modified the powder microstructure, promoting the formation of microporous, spongy textures that facilitated sugar removal and slowed down the hygroscopicity. CP-US-EZ-treated powder exhibited the lowest hygroscopicity and highest contact angle, indicating enhanced resistance to moisture uptake and hydrophobicity. This group showed the greatest content of total phenolics, flavonoids, vitamin C, antioxidant capacity, and monophenols, including 3,4-dihydroxybenzoic acid, protocatechualdehyde, and rutin. These findings demonstrate that CP-US-EZ is a promising non-thermal pretreatment technology to solve the hygroscopicity problem of the powder and produce high-quality, low-sugar jujube powder.

## Graphical abstract

**Figure:**
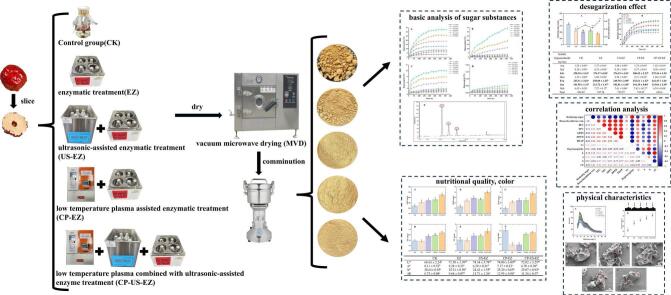

## 1 Introduction

*Ziziphus jujuba* Mill. cv. *Hamidazao*, a member of the Rhamnaceae family, has been cultivated in China for millennia and has recently gained popularity in Europe and North America due to its rich content of vitamin C, polyphenols, and polysaccharides [1]. Despite its nutritional value and health benefits, fresh jujube is highly perishable, which is not conducive to the realization of its proper economic value, prompting the widespread adoption of drying methods to extend shelf life and facilitate storage [2].

Jujube powder, a novel powdered fruit and vegetable product, offers convenience in consumption and versatility in processing. It is typically prepared through vacuum microwave drying (MVD), a method that leverages the benefits of both vacuum and microwave processing to significantly reduce drying time and better preserve the nutritional value and marketability of the dried product [1]. However, the high sugar content of jujube powder makes it highly susceptible to undesirable problems like the caking phenomenon and hygroscopicity during storage and transportation, which can negatively impact consumer perception and satisfaction. Currently, caking poses a significant challenge, limiting the edible quality and processing characteristics of jujube powder [3]. Therefore, there is an urgent need for the jujube powder processing industry to develop effective treatments to solve hygroscopicity and caking issues.

To address the caking issue in high-sugar fruit and vegetable powders, researchers often use anticaking agents such as silica, maltodextrin, tricalcium phosphate, and calcium silicate are commonly used. However, long-term or excessive consumption of these additives may pose health risks [4]. As a result, developing additive-free technologies to inhibit caking has become a priority in food processing research. Previous studies have shown a strong positive correlation between fruit and vegetable powder caking and the presence of reducing sugars, particularly glucose and fructose [5,6]. According to Liu et al. [7], dried jujube contains over 70 % saccharides, with glucose and fructose as the dominant components. Enzyme treatment (EZ) has been shown to reduce stickiness and enhance powder flowability, and it has been successfully applied in producing powders from honeydew melon [8], pumpkin [9], and carrot [10]. Glucose oxidase (GOD), an enzyme with high catalytic efficiency, specificity, and non-toxic characteristics, catalyzes the oxidation of glucose into gluconic acid and hydrogen peroxide [11]. Given the sugar composition of jujube and the catalytic properties of GOD, we hypothesized that enzymatic desugarization using GOD could be a potential strategy to mitigate agglomeration in jujube powder. However, the efficacy of standalone EZ is limited due to poor enzyme penetration and restricted saccharide removal in intact plant tissues. Therefore, it is necessary to integrate physical enhancement techniques to improve enzyme accessibility and effectiveness.

In recent years, non-thermal technologies have gained increasing attention in food processing as innovative pretreatment strategies. Techniques such as ultrasound (US), cold plasma (CP), pulsed electric field (PEF), and ultra-high pressure (UHP) have shown great potential. Among these, the US, an acoustic mechanical wave, exhibits strong penetrability. Its cavitation and sponge-like effects can change the native structure of fruit and vegetable tissues, thereby enhancing material quality and drying efficiency [12]. For example, Zang et al. [13] applied US as a drying pretreatment for cherries and observed reductions in bitterness and acidity, along with improved antioxidant activity and sensory acceptability. Similarly, Aydar et al. [12] demonstrated that US pretreatment of *Inula viscosa* (L.) before microwave drying increased the content of chlorophyll, carotenoids, total phenols, and antioxidants. Liu et al. [14] further reported that ultrasound-assisted enzyme treatment (US-EZ) significantly enhanced the extraction of anthocyanins from *Lycium ruthenicum*. Cold plasma (CP), another emerging non-thermal technology, generates reactive oxygen species, free radicals, and charged particles through gas ionization. These reactive components alter plant structure and have been successfully applied in enzyme inactivation, microbial control, drying enhancement, and pesticide degradation [15,16]. Zhou et al. [15] used CP to pretreat *Lycium barbarum* before hot air drying and found improved rehydration ability and phytochemical retention. CP also facilitated water migration by damaging the cell wall and cell structure of the fruit, helping to reduce drying time and preserve the original color of the fruit. In another study, Zhou et al. [17] showed that CP-treated cantaloupe better retained color and aroma and exhibited delayed ascorbic acid degradation. Furthermore, combining physical treatments has shown better advantages over individual approaches. Loukri et al. [18] found that combined US-CP pretreatment significantly increased total phenolics, loganic acid content, and DPPH radical scavenging activity compared to untreated cornelian cherry pomace. Li et al. [19] compared PEF, US, and combined PEF-US pretreatments for shiitake mushrooms and concluded that the combined approach resulted in shorter drying time, better color retention, higher phenolic content and nutrients. These findings underscore the potential of US and CP as pretreatments for improving the quality of dried products. Based on this evidence, we think that cold plasma synergistic ultrasound-assisted enzyme treatment (CP-US-EZ) could address the limitations of single treatments, improve hygroscopicity in jujube powder, and enhance the quality of the products.

In recent years, there have been few reports on the effects of EZ, US-EZ, CP-EZ, and CP-US-EZ on hygroscopicity as well as the quality of high-sugar fruit and vegetable powders. Therefore, in this study, to address the agglomeration issue in jujube powder, based on the sugar substances and hygroscopic causes, we analyzed and investigated: (1) the improvement of hygroscopicity of jujube powder by EZ, US-EZ, CP-EZ, and CP-US-EZ; (2) the differences in bioactive substances and antioxidant capacity caused by pretreatment; and (3) the differences in microstructure, hydrophilicity, and crystalline strength. The objective was to identify a suitable pretreatment to slow down the negative effects of hygroscopicity on jujube powder while retaining the nutrients and to provide a new approach to improve the phenomenon of fruit and vegetable powder agglomeration.

## 2 Materials and methods

### 2.1 Raw materials

*Z. jujuba* cv. *Hamidazao* was sourced from the jujube farms of the 13th Division of the Xinjiang Production and Construction Corps, China. Fruits without visible mechanical damage, pests, or diseases and uniform in size and color were selected. The jujubes were washed, pitted, and sliced into 11 ± 0.5 mm thick slices prior to pretreatment.

### 2.2 Pretreatment

Five groups were established:

(a) Enzyme treatment (EZ): Jujube slices were immersed in a glucose oxidase (GOD) solution (0.2 % w/v, material-to-liquid ratio of 1:3) at 45°C for 40 min.
(b) Ultrasound-assisted enzyme treatment (US-EZ): Slices were first sonicated in an ultrasonic bath (KQ-400DE, Kunshan Ultrasonic Instrument Co., Ltd., Jiangsu, China) at 240 W and 40 kHz for 45 min, followed by enzyme treatment under the same conditions as (a).
(c) Cold plasma-assisted enzyme treatment (CP-EZ): Slices were sealed in polyethylene bags and treated with cold plasma (PG-1000ZD, Nanjing Suman Plasma Science and Technology Co., Ltd., Jiangsu, China) at 150 kV for 300 s, then subjected to (a).
(d) Cold plasma synergistic ultrasound-assisted enzyme treatment (CP-US-EZ): Jujube slices were sequentially treated with cold plasma, ultrasound, and enzyme as described in (c), (b), and (a), respectively.
(e) Control group (CK): Slices were soaked in distilled water (1:3, w/v) at 45°C for 40 min without enzyme or physical treatment.

### 2.3 Vacuum microwave drying (MVD)

Pre-treated jujube slices were dewatered using an electric vegetable centrifuge, spread evenly on drying trays, and dried in a vacuum microwave dryer (RWBZ-08S, Nanjing Suenrui Drying Equipment Co., Ltd., Jiangsu, China) until the moisture content was below 8 %. The dried samples were ground into powder using a high-speed pulverizer (FW100, Beijing Kobai Science and Technology Co., Ltd., Beijing, China).

### 2.4 Analysis of sugar substances

#### 2.4.1 Reducing sugar and desugarization rate

Reducing sugar content was measured using a commercial reducing sugar assay kit (Suzhou Grace Biotechnology Co., Ltd., Jiangsu, China) by determining absorbance at 500 nm. A glucose standard curve was used to quantify results, expressed in mg glucose equivalent per gram of powder (mg/g).

The desugarization rate (*W*) was calculated using Eq. (1):

(1) $$W\left(\%\right)=\left(X_{1}-X_{2}\right)/X_{1}\times 100$$

where *X_1_* and *X_2_* represent the reducing sugar content before and after pretreatment, respectively.

#### 2.4.2 Soluble monosaccharides

Approximately 100 mg of jujube powder was extracted with 700 µL of 80 % ethanol at 50°C for 2 h, then diluted with 700 µL of deionized water and centrifuged at 10,000 × g for 3 min. The supernatant was collected for HPLC analysis.

HPLC was performed using a Thermo ICS 5000 + ion chromatography system (Thermo Fisher Scientific, USA) equipped with a CarboPac™ PA1 column (250 × 4.0 mm). The mobile phases were A: deionized water and B: 100 mM NaOH. The injection volume was 10 µL, the flow rate was 1.0 mL/min, and the column temperature was 30 °C. The gradient elution profile was 0 min (95:5, A:B), 12 min (90:10), 15 min (0:100), 25 min (0:100), 40 min (95:5), and 60 min (95:5).

#### 2.4.3 Hygroscopicity

Hygroscopicity was determined using the saturated salt solution method [20]. Samples (3 g) of sucrose, glucose, fructose, and jujube powder were weighed (*M_0_*), placed in weighing flasks (*M_1_*), and exposed to controlled humidity conditions (25°C) over saturated different salt solutions. Different saturated salt solutions provided different levels of relative humidity (RH) as follows: saturated lithium chloride solution (11.30 % RH), saturated potassium acetate solution (22.51 % RH), saturated magnesium chloride solution (32.78 % RH), saturated potassium carbonate solution (43.16 % RH), saturated sodium bromide (57.57 % RH), saturated potassium iodide (68.86 % RH) and saturated sodium chloride (75.29 % RH) [20]. Sample mass (*M_2_*) was recorded at 0, 3, 6, 12, 24 h, and every 24 h thereafter until equilibrium. Each sample was tested in triplicate, and the hygroscopicity curve was plotted with the sample hygroscopicity as the vertical coordinate and the hygroscopicity time as the horizontal coordinate. The hygroscopicity was plotted against time using Eq. (2).

(2) $$Hygroscopicity\left(\%\right)=\left(M_{2}-M_{1}-M_{0}\right)/M_{0}\times 100$$

### 2.5 Physical property characterization

#### 2.5.1 Scanning electron microscopy (SEM)

Ten milligrams of jujube powder was affixed to conductive tape and imaged using a field emission SEM (Quattro S, Thermo Fisher Scientific, USA) at 300 × magnification.

#### 2.5.2 X-ray diffraction (XRD)

The jujube powder was analyzed with an XRD diffractometer (Rigaku Ultima IV, Japan) with a scan range of 10-80° and a scanning rate of 10°/min.

#### 2.5.3 Contact angle measurement

The contact angle was measured using the seated drop method [21]. Powder tablets (15 mm diameter, approximately 1 mm thick) were pressed and analyzed using a contact angle goniometer (SZ-CAMC32, Shanghai Xuanzhun Instrument Co., Ltd., Shanghai, China). The drop profile was fitted to determine θ.

### 2.6 Nutritional quality assessment

#### 2.6.1 Total phenols (TPC) and total flavonoids (TFC)

The TPC content was measured by the Folin-Ciocalteu method and expressed as mg gallic acid equivalents per gram (mg/g). The TFC was determined by a colorimetric method and expressed as mg rutin equivalents per gram (mg/g) [7].

#### 2.6.2 Antioxidant capacity

Antioxidant activity was assessed using DPPH, ABTS, and FRAP kits (Suzhou Grace Biotechnology Co., Ltd., Jiangsu, China). Absorbance was recorded at 517 nm (DPPH), 734 nm (ABTS), and 590 nm (FRAP). Results were expressed as mg Trolox equivalent per gram (mg Trolox/g).

#### 2.6.3 Vitamin C (Vc)

Vc was determined by colorimetric titration using 2,6-dichlorophenol indophenol [1] and expressed as mg ascorbic acid per 100 g (mg/100 g) of jujube powder.

#### 2.6.4 Determination of phenolic compounds

Referring to the method of Zhang et al. [22] with slight modification, 0.5 mL of 80 % methanol (containing 0.2 % Vc) was added to each sample, vortexed, ultrasonicated for 30 min, and centrifuged at 12,000×*g* for 10 min. This process was repeated twice, and supernatants were combined. Analysis was performed via UPLC (Vanquish, Thermo Scientific, USA) and Q Exactive mass spectrometry (Thermo Scientific, USA).

Chromatographic conditions: Waters HSS T3 column (50 × 2.1 mm, 1.8 μm); mobile phases: A (water with 0.1 % formic acid), B (acetonitrile with 0.1 % formic acid); flow rate: 0.3 mL/min; temperature: 40 °C; injection volume: 2 µL. Gradient: 0–2 min (90:10 A: B), 2–6 min (90:10), 6–9 min (40:60), 9.1–12 min (90:10).

### 2.7 Color

Color attributes (L*, a*, b*) were measured using a portable colorimeter (YS3060, 3NH, Co., Ltd., Shanghai, China). L* indicates brightness, a* the red–green coordinate, and b* the yellow-blue coordinate. Color difference (Δ*E*) was calculated by the formula in Eq. (3).

(3) $$\Delta E=\sqrt{{\left(L*-L_{0}\right)}^{2}+{\left(a*-a_{0}\right)}^{2}+{\left(b*-b_{0}\right)}^{2}}$$

where: *L_0_, a_0_, and b_0_* are the color values of the control group.

### 2.8 Data analysis

All experiments were conducted in triplicate. Data were reported as mean ± standard error. Statistical significance was determined by one-way ANOVA using IBM SPSS Statistics 26.0 (IBM Corp., Armonk, NY, USA). Graphs were prepared using Origin 2021 software (OriginLab, Northampton, MA, USA). The value of *P* < 0.05 was statistically considered significant.

## 3 Results and discussion

### 3.1 Sugar substance basis

**Fig. 1:**
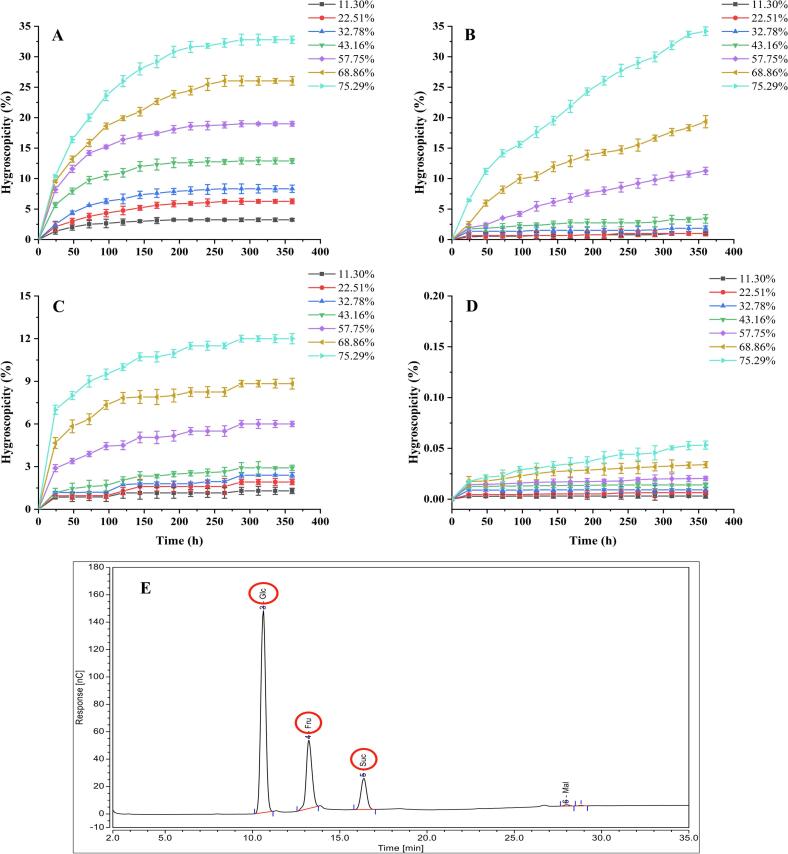
Hygroscopicity of jujube powder (A), fructose (B), glucose (C), and sucrose (D) under seven kinds of ambient relative humidity (RH); content of soluble monosaccharides in jujube powder (E).

To evaluate the relationship between the caking behavior of jujube powder and ambient relative humidity (RH), the present study firstly simulated seven controlled humidity environments (11.30 %, 22.51 %, 32.78 %, 43.16 %, 57.75 %, 68.86 %, and 75.29 %) using the saturated salt solution and observed the hygroscopicity pattern of jujube powder (Fig. 1A). Overall, the hygroscopicity curve of jujube powder showed an increasing and then stabilizing trend with the extension of time. At RH 75.29 %, the jujube powder exhibited a hygroscopicity of 32.80 %, which was 10.12 times higher than that observed at RH 11.30 %. For comparison, Oh et al. [23] reported that sweet potato powder exhibited only 13 % hygroscopicity at the same RH level, indicating that jujube powder is particularly susceptible to moisture uptake under humid conditions.

As previously reported, the hygroscopicity of plant-based powders correlates with their sugar content [6]. HPLC analysis (Table S1) revealed that glucose (359.25 µg/mg) and fructose (324.33 µg/mg) were the predominant sugars in jujube powder, followed by sucrose (140.89 µg/mg), with the total saccharide content exceeding 80 % (Fig. 1E). To further explore the role of specific saccharides, the hygroscopicity of individual sugars (fructose, glucose, and sucrose) was examined under the same RH conditions (Fig. 1B–D). The results showed that the hygroscopicity of fructose at high RH (57.75 %–75.29 %) was positively correlated with RH. Fructose demonstrated the highest hygroscopicity, reaching 34.19 % at RH 75.29 %, while glucose showed a moderate level (12.00 %), and sucrose was almost hygroscopic. This is related to the fact that fructose and glucose, as soluble monosaccharides with low glass transition temperatures (Tg) and multiple hydroxyl groups, significantly reduce Tg and promote hygroscopicity in sugar-rich materials [5], which causes the product to become hygroscopic and sticky. It also implies that fructose and glucose within the jujube powder may be the substances responsible for the extreme hygroscopicity of the jujube powder. Furthermore, the Pearson's index (Table 1) confirmed this relationship: fructose and glucose contents were positively correlated with jujube powder hygroscopicity (r = 0.86 and r = 0.46, respectively), while sucrose showed a negative correlation (r = −0.78). These findings support the hypothesis that reducing sugars, particularly fructose and glucose, are key contributors to the hygroscopic agglomeration of fruit and vegetable powders.

**Table 1:** Correlation coefficients between the three sugar contents and the hygroscopicity of jujube powder.

Parameters	Moisture absorption (%)	Glucose (ug/mg)	Fructose (ug/mg)	Sucrose (ug/mg)
Moisture absorption (%)	1	**0.46**	**0.86**	−0.78
Glucose (ug/mg)	**0.46**	1	0.59	−0.42
Fructose (ug/mg)	**0.86**	0.59	1	−0.82
Sucrose (ug/mg)	−0.78	−0.42	−0.82	1

### 3.2 Soluble monosaccharides

**Fig. 2:**
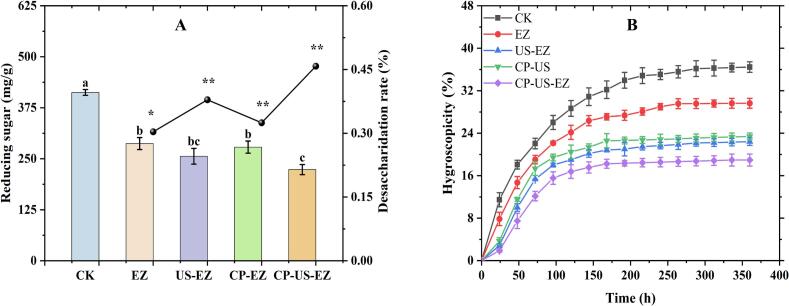
Reducing sugar content, desugarization rate (A), and hygroscopicity (B) of jujube powders with different pretreatments. *: *P* < 0.05 significant level; **: *P* < 0.01 extremely significant level. Different lowercase letters in the same column indicate significant differences, *P* < 0.05.

**Fig. 3:**
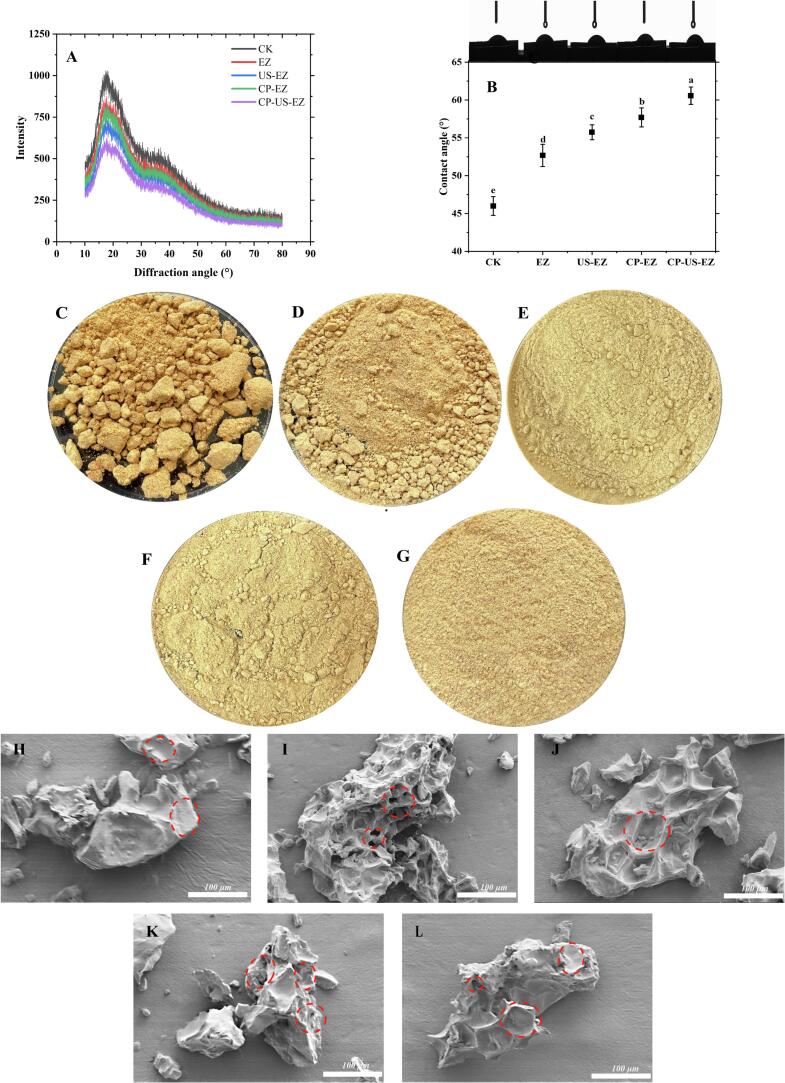
XRD (A), contact angle (B), appearance (C-G; 300 × ), and SEM (H-L) of jujube powder with different pretreatments.

Table 2 presents the soluble monosaccharide composition of jujube powder following various pretreatments. Glucose, fructose, and sucrose remained the predominant sugars across all groups, followed by smaller amounts of maltose, mannan, and arabinose. Pretreated samples exhibited a noticeable reduction in glucose and fructose levels compared to the control (CK). Among the treatment groups, the difference in glucose content was not statistically significant between the EZ, US-EZ, and CP-US-EZ groups. Notably, the CP-US-EZ group showed a substantial reduction in fructose content (242.47 μg/mg), which was 25.42 % lower than the CK group and 3.01 %, 2.93 %, and 4.01 % lower than the EZ, US-EZ, and CP-EZ groups, respectively. On the one hand, this notable decrease in fructose may be attributed to the action of cold plasma (CP), which generates free radicals and reactive substances to react with fructose molecules, leading to structural changes and degradation [24]. On the other hand, ultrasound (US) and vacuum microwave drying (MVD) high-frequency vibrations can generate strong shear force, contributing to structural disruption and enhanced fructose degradation [12]. These findings confirm the feasibility of the non-thermal technique in combination with enzyme treatment. In addition, glucose and fructose, as substrates for the Maillard reaction, are continuously consumed during the drying process [7]. Interestingly, sucrose content increased in all treatment groups except CP-US-EZ. This could be due to the condensation of glucose and fructose into sucrose, facilitated by sucrose synthase activity, and these monosaccharides are more abundant and have a higher probability of contacting each other, which accelerates the enzymatic reaction [25]. In conjunction with the microstructure (Fig. 3H–L), this supports the idea that the formation of cell wall microchannels may enhance the interaction of glucose and fructose. In this study, the total soluble monosaccharide content followed the descending order: CK > EZ > US-EZ > CP-EZ > CP-US-EZ. This trend was generally similar to that of hygroscopicity (Fig. 2B), reinforcing the correlation between high monosaccharide content and hygroscopicity. As reported by Feng et al. [6], polysaccharides tend to suppress hygroscopicity, whereas mono- and disaccharides promote it, consistent with the present findings. Overall, the CP-US-EZ pretreatment proved most effective in reducing soluble monosaccharides, particularly glucose and fructose, which are key contributors to the hygroscopic caking phenomenon.

**Table 2:** Effects of different pretreatments on soluble monosaccharides in jujube powder.

Soluble monosaccharides (ug/mg)	CK	EZ	US-EZ	CP-EZ	CP-US-EZ
Ara	1.52 ± 0.01^b^	1.27 ± 0.01^c^	1.48 ± 0.01^b^	1.23 ± 0.01^d^	1.62 ± 0.01^a^
Gal	0.28 ± 0.01^a^	0.23 ± 0.01^b^	0.20 ± 0.01^c^	0.27 ± 0.01^a^	0.20 ± 0.01^c^
**Glc**	**358.10 ± 2.63^a^**	**274.17 ± 3.83^c^**	**276.15 ± 4.26^c^**	**280.62 ± 2.23^b^**	**275.16 ± 3.26^c^**
Man	1.32 ± 0.02^e^	1.88 ± 0.02^c^	2.94 ± 0.01^a^	2.31 ± 0.01^b^	1.60 ± 0.10^d^
**Fru**	**325.11 ± 3.82^a^**	**250.00 ± 1.25^b^**	**249.78 ± 2.90^b^**	**252.61 ± 1.33^b^**	**242.47 ± 1.61^c^**
**Suc**	**141.50 ± 1.31^d^**	**212.72 ± 1.67^a^**	**182.81 ± 1.68^b^**	**166.39 ± 0.84^c^**	**133.01 ± 2.45^e^**
Mal	6.62 ± 0.20^c^	7.27 ± 0.17^b^	7.61 ± 0.08^a^	7.42 ± 0.12^ab^	6.54 ± 0.08^c^
Total	834.45	747.54	720.97	710.85	660.6

*Note*: Different lowercase letters in the same row indicate significant differences, *P* < 0.05.

### 3.3 Desugarization rate

To solve the problem of caking of jujube powder caused by the hygroscopicity of reducing sugars such as glucose and fructose, four pretreatment strategies (EZ, US-EZ, CP-EZ, and CP-US-EZ) of desugarization were applied prior to MVD. As shown in Fig. 2A, all pretreatments effectively reduced the reducing sugar content compared to the CK. The EZ group achieved a desugarization rate of 30.33 %, while US- and CP-assisted enzyme treatments further enhanced sugar removal. Among them, the CP-US-EZ group exhibited the highest desugarization rate at 45.80 %. This reduction is consistent with the glucose and fructose content trends observed in Table 2. According to the US and CP mechanism, this can be attributed to their ability to disrupt cellular microstructure, thereby improving enzyme penetration, facilitating the diffusion of glucose oxidase (GOD) into the cellular matrix, and giving GOD more opportunity to catalyze the conversion of glucose to gluconic acid [11]. In addition, Laika et al. [26] reported that CP can produce high-energy electrons, free radicals, and UV rays that can disrupt the chemical bonds of fructose (e.g., C–O, C–C bonds), leading to fructose degradation.

The hygroscopicity of the treated jujube powder samples (RH 75.29 %) is shown in Fig. 2B, where all the treated samples showed lower hygroscopicity than CK, with the CP-US-EZ group showing the lowest hygroscopicity, followed by the US-EZ, CP-EZ, and EZ groups. This suggests that the partial removal of sugar is beneficial in slowing down the hygroscopicity of the powder, which reaffirms the conclusion that sugar is a key factor in causing agglomeration of jujube powders. The fact that CP-US-EZ showed significant anti-caking ability also implies that there is some kind of synergistic effect between CP and US. In conclusion, we concluded that CP-US-EZ is an efficient, sugar-reducing, and moisture-reducing pretreatment for jujube powder.

### 3.4 Physical properties

#### 3.4.1 Crystalline strength

The diffraction peaks of XRD are commonly used to reflect the degree of powder agglomeration, and the results are shown in Fig. 3A. The control group (CK) exhibited the highest diffraction peak intensity (1029.82), followed by EZ, CP-EZ, and US-EZ (861.32, 826.29, and 745.70), and the smallest peak was observed for the CP-US-EZ group (626.75). This decline in peak intensity reflects a reduction in crystallinity, which is associated with decreased powder caking. Combined with the SEM results, the CK group had the highest crystallization peaks due to the dense structure; the sugar substance did not transform or degrade, and it was easy to attach to the surface of the dry matter to form a hard shell. In contrast, pretreatment groups exhibited varying degrees of microstructural disruption, and the peaks all showed a decreasing trend. From Fig. 3C–G, the appearance and morphology of the different pretreated jujube powders can be observed, and the CK group (Fig. 3C) had a serious and large number of agglomerations, which is consistent with the XRD results. The agglomerations of jujube powder were reduced to different degrees after treatment, in which the EZ group (Fig. 3D) still had irregular and uneven lumps, while the US-EZ (Fig. 3E) and CP-EZ (Fig. 3F) groups demonstrated improved powder flowability but retained small agglomerates. The CP-US-EZ group (Fig. 3G), which had the lowest XRD peak, exhibited the best powder fluidity and no agglomeration, which verifies the accuracy of the conclusions of this study. The effectiveness of CP-US-EZ in reducing crystallinity can be attributed to its ability to cause structural damage to plant tissue, which facilitates enzyme penetration and promotes water molecule entry, and affects the flexibility of the material itself, and the combined treatments disrupted the hydrogen bonding with greater strength, reducing the crystalline zone of the jujube powder and lowering the diffraction peaks and the crystalline strength [27]. According to Davoudi et al. [28], CP disrupts the crystalline domains by targeting hydrogen bonds within the matrix, thereby loosening the structure and enhancing enzymatic accessibility. Similarly, Wang et al. [29] reported that US weakens intermolecular interactions in plant matrices, promoting chain disentanglement and increasing structural disorder, which also contributes to reduced crystallinity. In conclusion, the CP-US-EZ pretreatment effectively improves jujube powder agglomeration by reducing the degree of powder crystallization.

#### 3.4.2 Contact angle

The contact angle serves as a visual and quantitative indicator of a powder's surface affinity to moisture and is commonly used to evaluate hygroscopicity in powder-based products [21]. Typically, contact angle and hydrophilicity are inversely related: a lower contact angle indicates stronger hydrophilicity, while a higher contact angle reflects increased hydrophobicity [30]. As shown in Fig. 3B, the CK group exhibited a contact angle of 45.99°. All pretreated groups showed significantly higher contact angles than the CK group, which suggests that the untreated sample has strong hydrophilicity and the pretreatments can reduce the hydrophilicity of the samples. Notably, the CP-US-EZ group had the highest contact angle (60.57°), representing a 31.70 % increase in hydrophobicity compared to CK. In comparison, the EZ, US-EZ, and CP-EZ groups demonstrated hydrophobicity increases of 14.55 %, 21.18 %, and 25.44 %, respectively. This improvement in hydrophobicity can be attributed to the more porous and regular microstructure formed after pretreatment, so that when a water droplet falls on the surface of the sample, the sample cannot absorb the water rapidly due to tension, increasing the contact angle [21]. Zhu et al. [30] also noted that the solid–liquid contact angle is influenced by the pull-off force, while physical treatment, chemical modification, and mechanical treatment all affect the surface properties. When a solid–liquid interface is formed between a liquid and a solid, the volume of the liquid increases, the pull-off force due to aggregation effects increases, which in turn increases the surface tension, and the contact angle increases significantly. These findings confirm that pretreatments, particularly CP-US-EZ, could reduce hygroscopicity by enhancing surface hydrophobicity.

#### 3.4.3 Microstructure

Improvement in dried product quality is usually closely related to microstructural changes [31]. To analyze the effects of various pretreatments, particularly the combined CP-US-EZ method, we examined the microstructure of jujube powder using SEM (Fig. 3H–L). In the control group (CK, Fig. 3H), the samples exhibited a smooth surface and compact structure. In contrast, the EZ-treated group (Fig. 3I) displayed varying numbers and sizes of pores, likely resulting from enzyme solution infiltration and the formation of fusion channels within the cell matrix [8]. The US-EZ group (Fig. 3J) showed a pronounced spongy texture, attributed to the cavitation effect of ultrasound, which causes repeated expansion, contraction, and stretching of the tissue [32]. In the CP-EZ group (Fig. 3K), the surface appeared heterogeneous, with both rough and smooth areas accompanied by irregular collapses, cracks, and micropores. This may be related to the breaking of chemical bonds and the disintegration of microscopic features of the material due to the etching effect of CP during the discharge process [28]. Interestingly, the CP-US-EZ group (Fig. 3L) exhibited spongy structures, cracks, and pores, but the complete structure is not unobservable as we expected; instead, the CP-US-EZ-treated samples showed only mild erosion and retained a relatively smooth surface, suggesting that this combined treatment caused less microstructural damage overall. In conclusion, we believe that several pretreatments can increase the permeability of the enzyme solution to the dry material by altering the microstructure of the sample, which validates our previous speculation.

### 3.5 Antioxidant capacity and bioactive substances

#### 3.5.1 Antioxidant capacity

**Fig. 4:**
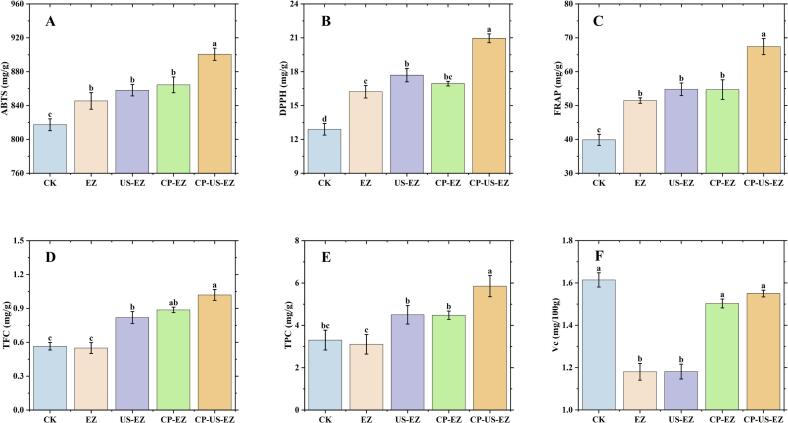
Antioxidant capacity (ABTS, DPPH, FARP) of jujube powder (A-C), TFC (D), TPC (E), and Vc (F) with different pretreatments. Different lowercase letters in the same column indicate significant differences, *P* < 0.05.

Antioxidant capacity is a key indicator for evaluating the functional properties and nutritional quality of food products [13]. In this study, the antioxidant activities of jujube powders subjected to different pretreatments were assessed using DPPH, ABTS, and FRAP assays (Fig. 4A–C). All pretreatment methods significantly enhanced antioxidant capacity compared to the untreated control. The ABTS (900.60 mg/g), DPPH (20.96 mg/g), and FRAP (67.42 mg/g) of CP-US-EZ in the treated group were higher than the rest of the samples. These findings are consistent with those of Bao et al. [24], who reported that CP-pretreated dried jujubes released active gases, and the antioxidant capacity of dried jujubes could be increased by 36.85 % through the etched microchannels. Similarly, Wang et al. [33] demonstrated that combining ultrasound with enzymatic treatment in noni juice enhanced phenolic content and antioxidant activity. The cavitation effects generated by ultrasound synergized with enzymatic reactions to increase the levels of DPPH and ABTS radical scavenging activity. Unlike conventional heat treatments, which often degrade antioxidants, CP-US-EZ employs non-thermal mechanisms to enhance the release of bioactives, which in turn improve the antioxidant capacity of the samples. In summary, CP-US-EZ pretreatment significantly improves the antioxidant capacity of jujube powder while simultaneously mitigating hygroscopicity, making it a promising approach for developing high-quality, functional powdered fruit products.

#### 3.5.2 TFC and TPC

Phenolics and flavonoids are important bioactive constituents of jujube [1]. In this study, the TFC and TPC of jujube powders subjected to different pretreatments were quantified (Fig. 4D and E). Compared with the CK (0.56 mg/g and 3.30 mg/g), the EZ treatment did not change significantly in TFC (0.55 mg/g) and TPC (3.11 mg/g). However, pretreatments involving US and CP significantly enhanced both TFC and TPC, with the highest TFC (1.02 mg/g) and TPC (5.85 mg/g) in the CP-US-EZ group. This enhancement may be attributed to the formation of micropores on the cell surface induced by US, CP, and EZ treatments, which facilitate water diffusion and reduce drying time. A shorter drying time favors the preservation of heat-sensitive bioactives [34]. Similar findings were reported by Yuan et al. [31] and Yazdi et al. [35], who noted that non-thermal treatments can induce depolymerization or polymerization of flavonoids, altering their molecular structure and enhancing bioactivity. Moreover, US and CP disrupt the cell wall, promoting the release of intracellular phenolic and flavonoid compounds.

**Fig. 5:**
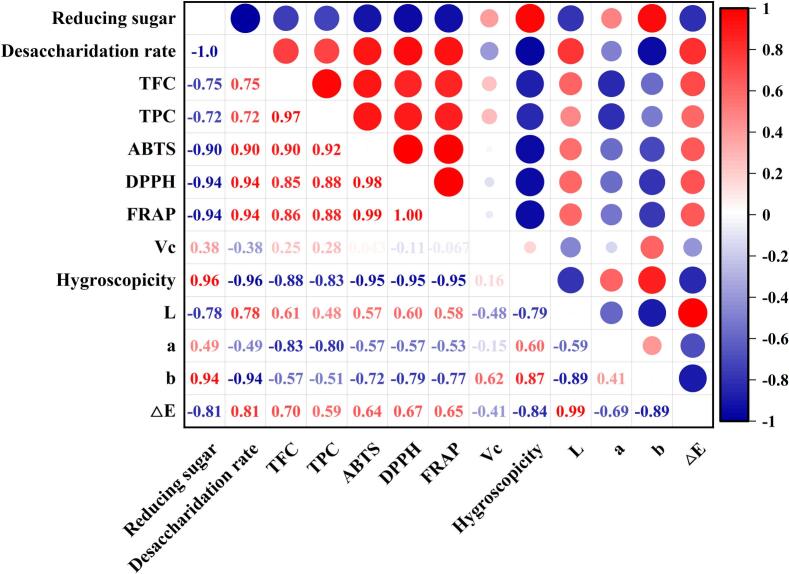
Correlation analysis between jujube powder multi-indicator of different pretreatments.

Correlation analysis (Fig. 5) revealed strong positive associations between antioxidant capacity (ABTS, DPPH, FRAP) and bioactives (TFC and TPC), with correlation coefficients ranging from 0.85 to 0.92. Notably, phenolics showed the highest correlation with antioxidant activity, supporting our previous speculation that appropriate US and CP treatments may promote the release of bioactives from jujube powder. These findings are consistent with Zang et al. [36], who demonstrated that combined ultrasonic and ultra-high-pressure pretreatment improved the retention of phenolics and flavonoids in peach slices during radiofrequency vacuum drying, and the trend of antioxidant capacity of the slices was similar to that of phenolics and flavonoids content. Based on this evidence, further analysis of monophenolic compounds was conducted to validate the contribution of specific bioactives to support our conclusions.

#### 3.5.3 Phenolic compounds

Phenolic compounds are widely recognized as the primary contributors to the antioxidant and potential anticancer activities of jujube powder [31]. To evaluate the impact of pretreatments on phenolic profiles, HPLC analysis was employed for the qualitative and quantitative determination of monomeric phenolics across the different treatment groups. A total of 26 individual phenolic compounds were identified, including gallic acid, 3,4-dihydroxybenzoic acid, vanillic acid, benzoic acid, and rutin (Table 3). This result aligns with the findings of Liu et al. [7], who reported rutin, salicylic acid, and benzoic acid as predominant phenolics in jujube powder across different drying methods. Following pretreatment, the overall trend showed a decrease in certain phenolics (e.g., benzoic acid), while most other phenolics increased in concentration. In terms of total content, the CP-US-EZ group exhibited the highest content (5670.52 μg/g), followed by the US-EZ group (4435.19 μg/g) and the CP-EZ group (4329.70 μg/g). This trend was consistent with the observed increase in total phenolic content (TPC), which may be due to the catalytic hydrolysis of bound phenols by reaction products such as hydrogen peroxide generated during the oxidation reaction of glucose, thereby converting them to free phenols, thus increasing the detection of monomeric phenols [16]. In addition, the free radicals generated by US may interact with the aromatic rings of phenolic compounds, thereby enhancing the antioxidant capacity of the bioactives. At the same time, pretreatment may disrupt covalent bonds, leading to cellular release of antioxidants such as phenols, flavonoids, and carotenoids [13]. In summary, we found that the CP-US-EZ treatment was the most advantageous in terms of TFC content, TPC content, and monomeric phenolic compounds.

**Table 3:** Effect of different pretreatments on phenolics in jujube powder.

Phenolic substance (ug/g)	CK	EZ	US-EZ	CP-EZ	CP-US-EZ
Gallic acid	3.10 ± 0.36^c^	3.12 ± 0.25^c^	5.77 ± 0.41^b^	6.89 ± 0.51^a^	3.58 ± 0.36^c^
3,4-Dihydroxybenzoic acid	71.33 ± 3.82^b^	70.03 ± 4.90^b^	96.28 ± 9.19^ab^	113.96 ± 7.15^a^	100.75 ± 9.15^b^
Protocatechualdehyde	30.67 ± 3.03^c^	35.46 ± 0.50^ab^	46.34 ± 0.31^ab^	53.47 ± 3.09^a^	46.34 ± 5.72^bc^
4-Hydroxybenzoic acid	59.62 ± 5.40^a^	48.01 ± 3.27^a^	75.54 ± 0.41^a^	76.64 ± 5.13^a^	84.12 ± 21.99^a^
Phthalic acid	11.29 ± 1.84^b^	11.16 ± 0.26^b^	12.46 ± 0.61^b^	22.95 ± 1.02^b^	303.04 ± 41.16^a^
Catechin	4.65 ± 0.18^b^	7.25 ± 0.34^a^	10.37 ± 0.51^a^	10.33 ± 0.51^a^	9.91 ± 1.03^a^
Vanillic acid	74.38 ± 4.59^bc^	62.38 ± 3.31^c^	98.37 ± 7.16^ab^	106.90 ± 6.18^a^	96.02 ± 6.96^bc^
Caffeic acid	9.03 ± 0.18^ab^	5.81 ± 0.16^c^	10.80 ± 0.42^a^	9.40 ± 0.51^ab^	9.71 ± 1.15^bc^
Syringic acid	14.08 ± 0.47^a^	11.22 ± 0.33^a^	16.59 ± 0.72^a^	17.87 ± 0.31^a^	16.73 ± 4.57^a^
Epicatechi	5.24 ± 0.28^c^	7.93 ± 0.57^ab^	11.82 ± 0.62^a^	8.74 ± 0.57^b^	11.32 ± 1.26^ab^
Vanillin	7.45 ± 0.46^a^	6.22 ± 0.33^b^	9.06 ± 0.82^ab^	10.37 ± 0.51^a^	9.64 ± 0.57^b^
p-Hydroxycinnamic Acid	31.14 ± 0.27^c^	23.06 ± 2.45^a^	37.43 ± 2.04^a^	38.89 ± 5.11^a^	42.64 ± 4.00^a^
Syringaldehyde	5.32 ± 0.36^b^	8.43 ± 0.49^a^	10.52 ± 0.61^a^	11.60 ± 1.33^a^	10.63 ± 1.60^a^
Rutin	1482.5 ± 55.21^d^	1740.82 ± 57.17^c^	2482.72 ± 82.38^b^	2184.53 ± 91.95^c^	3177.89 ± 115.99^a^
Trans-Ferulic acid	10.19 ± 0.73^ab^	10.01 ± 0.49^ab^	15.76 ± 2.56^a^	11.21 ± 1.02^ab^	10.79 ± 1.49^b^
Sinapic Aci	31.14 ± 0.27^a^	19.94 ± 6.53^a^	39.93 ± 4.08^a^	41.34 ± 3.06^a^	37.77 ± 8.00^a^
Salicylic acid	228.53 ± 7.20^ab^	158.60 ± 6.59^b^	251.96 ± 40.92^ab^	306.17 ± 25.52^a^	276.96 ± 32.01^ab^
Quercetin 3-β-D-glucoside	14.60 ± 0.45^a^	14.91 ± 0.57^a^	22.44 ± 4.08^a^	18.24 ± 2.56^a^	28.97 ± 8.03^a^
(+)-Dihydroquercetin	1.99 ± 0.18^b^	2.61 ± 0.49^ab^	3.87 ± 0.51^ab^	4.02 ± 0.41^a^	4.05 ± 0.80^ab^
Benzoic acid	924.83 ± 53.90^a^	583.14 ± 65.47^b^	848.36 ± 53.29^ab^	903.58 ± 76.64^ab^	997.11 ± 57.32^ab^
Kaempferol-3-O-glucoside	3.32 ± 0.54^b^	4.53 ± 0.25^a^	5.93 ± 0.73^a^	5.91 ± 0.72^a^	6.52 ± 0.80^a^
Quercetin	10.55 ± 0.47^a^	10.48 ± 2.04^a^	18.43 ± 5.12^a^	13.27 ± 2.56^a^	15.75 ± 3.46^a^
Hydrocinnamic acid	171.77 ± 5.39^ab^	129.07 ± 7.35^b^	210.74 ± 28.60^ab^	238.90 ± 26.42^a^	244.67 ± 10.30^ab^
Trans-Cinnamic acid	44.80 ± 4.51^ab^	36.06 ± 1.64^b^	61.58 ± 5.12^a^	60.26 ± 6.14^a^	69.99 ± 2.30^a^
Kaempferol	2.48 ± 0.65^a^	6.20 ± 5.52^a^	3.34 ± 0.51^a^	3.34 ± 0.31^a^	3.54 ± 0.57^a^
Gossypol	27.75 ± 6.30^bc^	25.32 ± 4.11^bc^	28.72 ± 3.06^c^	50.89 ± 5.13^a^	52.07 ± 5.73^ab^
Total	3281.75	3041.79	4435.19	4329.70	5670.52

*Note*: Different lowercase letters in the same row indicate significant differences, *P* < 0.05.

### 3.6 Vc

Vc is highly sensitive to degradation during processing, particularly due to exposure to oxygen, enzymatic activity, and elevated temperatures during drying [32]. The Vc content of jujube powders subjected to different pretreatments is shown in Fig. 4F. Among all groups, the CP-EZ (93.17 %) and CP-US-EZ (96.27 %) treatments achieved the highest Vc retention, with no significant difference between them, while lower retention levels were observed in the EZ and US-EZ groups. These results are consistent with previous studies. Giannoglou et al. [37] demonstrated that CP treatment enhances cell membrane permeability, thereby increasing the extractability and preservation of ascorbic acid in dried strawberries. Similarly, Li et al. [32] reported that Vc retention in freeze-dried apricots was significantly higher in co-treated samples compared to those subjected to US alone. Wu et al. [38] also observed that while US treatment could protect Vc in pineapple nectar, it may simultaneously promote Vc degradation when applied alone. The differential outcomes observed in this study may be attributed to the following factors: (a) The formation of microporous structures in the EZ- and US-EZ-treated jujube powders increases the exposure of Vc to degradative enzymes such as ascorbate oxidase and ascorbate peroxidase, resulting in accelerated Vc loss [32]; (b) The addition of CP leads to the formation of surface cracks, enhancing water migration and significantly reducing the drying time. This shortening contributes to improved Vc preservation. In conclusion, the CP-US-EZ pretreatment not only effectively mitigated the caking tendency of jujube powder but also promoted the retention of Vc, thereby improving the product’s nutritional value.

### 3.7 Color

Color is a key quality attribute that significantly influences consumer perception and acceptability of fruit and vegetable products, which can provide preliminary information for people's preference or consumption [36]. The color parameters (L*, a*, b*, and *ΔE*) of jujube powders subjected to different pretreatments are presented in Table 4. Compared to the CK, all pretreatments resulted in increased L* values and decreased b* values, suggesting a general enhancement in brightness and a reduction in yellowness. However, these changes were not statistically significant. This trend is in agreement with Wu et al. [38], who observed that ultrasonic pretreatment did not cause significant color alterations in IR radiation-dried pineapple nectar. The increase in L* values can be attributed to reduced browning, which is likely linked to a decrease in reducing sugar content that limits Maillard reaction intensity. This also supports the hypothesis that pretreatment reduces reducing sugars. The reduction in b* values may be due to a decrease in carotenoid levels caused by temperature fluctuations during the drying process [38]. These findings further suggest that the pretreatment methods inhibit the browning reaction, thereby preserving color integrity. A consistent trend was observed by Li et al. [32] in dried apricot slices, where US, freeze–thaw (AT), and AT-US pretreatments increased L* values and decreased b* values.

**Table 4:** Color parameters of different pretreated jujube powders.

	CK	EZ	US-EZ	CP-EZ	CP-US-EZ
L*	68.63 ± 2.24^b^	71.50 ± 1.99^ab^	74.34 ± 5.78^ab^	74.00 ± 3.09^ab^	72.02 ± 3.29^ab^
a*	8.11 ± 0.52^b^	9.28 ± 0.35^a^	6.29 ± 0.31^d^	7.17 ± 0.23^c^	6.70 ± 0.30^d^
b*	28.61 ± 0.95^a^	25.31 ± 0.50^b^	24.42 ± 1.55^b^	25.29 ± 0.65^b^	25.07 ± 0.93^b^
ΔE	6.73 ± 0.08^e^	9.64 ± 0.07^d^	13.71 ± 1.26^a^	12.95 ± 0.96^b^	11.34 ± 0.37^c^

*Note*: Different lowercase letters in the same row indicate significant differences, *P* < 0.05.

In the present study, the a* values decreased in all treatment groups except EZ, with the most notable reduction observed in the US-EZ group, which may be related to the non-enzymatic pigment degradation induced by surface temperature. We hypothesize that the effect of pretreatment on color is related to both cell membrane rupture and structural changes, as these changes result in changes in internal scattered light and surface reflections, making it easier to eliminate pigments from plant tissues [31]. All pretreatments produced color differences (*ΔE*). The US-EZ and CP-EZ groups exhibited the most prominent variations (*ΔE* of 13.71 and 12.95, respectively), whereas the EZ and CP-US-EZ groups demonstrated smaller color deviations, which indicates that CP-US-EZ (*ΔE* of 11.34) has superior color retention compared to a single treatment. In summary, considering all color metrics, CP-US-EZ treatment demonstrated the most effective preservation of the original color characteristics of jujube powder.

### 3.8 Multi-indicator correlation analysis

To further investigate the relationship between desugarization pretreatments (EZ, US-EZ, CP-EZ, and CP-US-EZ) and various quality indicators of jujube powder, a multi-indicator correlation analysis was conducted. As shown by the Pearson correlation analysis in Fig. 5, red hues indicate positive correlations (0 < r < 1), blue hues indicate negative correlations (−1 < r < 0), and the intensity of color reflects the strength of the correlation. Hygroscopicity exhibited a strong positive correlation with reducing sugar content (r = 0.96), indicating that higher reducing sugar levels significantly increase the hygroscopicity of jujube powder. This supports earlier findings that monosaccharides, particularly glucose and fructose, are major contributors to hygroscopicity and caking. Using desugarization rate as a key index, pretreatment was found to positively influence several nutritional and functional parameters, including TFC, TPC, ABTS, DPPH, FRAP, and L* value, with correlation coefficients of 0.75, 0.72, 0.90, 0.94, 0.94, and 0.78, respectively. These results demonstrate that desugarization improves antioxidant activity and enhances the brightness of the powder. Conversely, negative correlations were observed between desugarization and Vc content (r = −0.38), a* value (r = −0.49), and b* value (r = −0.94), suggesting that although desugarization improves antioxidant profiles and color lightness, it may lead to partial degradation of ascorbic acid and minor shifts in color. Overall, these correlations are consistent with prior analyses and confirm that combined pretreatments not only reduce hygroscopicity but also enhance the functional and visual quality of jujube powder.

## 4 Conclusions

To address the challenges of hygroscopicity and caking in jujube powder and improve its overall quality, this study systematically analyzed the sugar composition and the causes of the jujube powder. Four pretreatment strategies (EZ, US-EZ, CP-EZ, and CP-US-EZ) were applied prior to MVD to explore the desugarization effect, nutritional quality, and physical properties of jujube powder. The results demonstrated that fructose and glucose were the primary contributors to the powder’s hygroscopicity. All pretreatments altered the cellular structure of jujube powder to different degrees, leading to the formation of micropores and spongy textures that facilitated enzyme penetration. This structural modification enhanced the degradation of reducing sugars and soluble monosaccharides, particularly glucose and fructose, thereby reducing the hydrophilicity and hygroscopicity of the final product. Among the treatments, CP-US-EZ was the most effective, resulting in the highest levels of bioactive compounds (TPC, TFC, and monomeric phenolics), superior antioxidant capacity (DPPH, ABTS, and FRAP), and the greatest retention of Vc (96.27 %). Importantly, these improvements were achieved without significant alterations in the color of the powder.

In summary, cold plasma synergistic ultrasound-assisted enzyme pretreatment offers a promising non-thermal approach to enhance the physicochemical and nutritional quality of jujube powder while mitigating its hygroscopicity. This strategy holds considerable potential for industrial-scale production and the development of high-quality, stable jujube-based products.

## CRediT authorship contribution statement

**Chenlu Zeng:** Writing – original draft, Methodology, Investigation, Conceptualization. **Yuxing Liu:** Methodology, Investigation. **Hao Dong:** Software, Data curation. **Yaxuan Liao:** Formal analysis, Data curation. **Ziyi Wang:** Validation, Formal analysis. **Wenjing Yang:** Validation, Formal analysis. **Jinling Lei:** Validation, Formal analysis. **Guogang Chen:** Validation, Supervision, Project administration, Conceptualization. **Wanting Yang:** Validation, Formal analysis. **Shaobo Cheng:** Writing – review & editing, Supervision, Resources, Funding acquisition, Formal analysis, Conceptualization.

## Declaration of competing interest

The authors declare that they have no known competing financial interests or personal relationships that could have appeared to influence the work reported in this paper.
