# Supplementary material for: Effect of cold plasma synergistic ultrasound-assisted enzyme treatment on the improvement of hygroscopicity and quality characteristics of jujube powder
Source: Ultrason Sonochem. 2025 Sep 11;121:107568. doi: 10.1016/j.ultsonch.2025.107568 (PMC12504961; doi:10.1016/j.ultsonch.2025.107568)
Supplement: Supplementary Data 1 [file mmc1.docx]

**Table S1**

Content of soluble monosaccharides in jujube powder

| Parameters | Glucose (ug/mg) | Fructose (ug/mg) | Sucrose (ug/mg) | Maltose (ug/mg) |
| --- | --- | --- | --- | --- |
| Jujube powder | 359.25 | 324.33 | 140.89 | 6.30 |
